# Screening instruments for antenatal and postpartum mental health disorders in migrant women: a systematic review

**DOI:** 10.1007/s00737-024-01552-z

**Published:** 2025-03-05

**Authors:** A. E. H. Verschuuren, E. Soldati, J. Stekelenburg, E. I. Feijen-de Jong, I. R. Postma

**Affiliations:** 1 Department of Health Sciences, Global Health Unit, University Medical Centre Groningen & University of Groningen, Groningen, The Netherlands; 2 Department of Psychiatry, Onze Lieve Vrouwe Gasthuis (OLVG), Amsterdam, The Netherlands; 3 Department of Obstetrics and Gynecology, Medical Center Leeuwarden, Leeuwarden, The Netherlands; 4 Department of Primary Care and Longterm Care, University Medical Centre Groningen & University of Groningen, Groningen, The Netherlands; 5 Department of Midwifery Science, Amsterdam University Medical Center Location Vrije Universiteit Amsterdam, Amsterdam, The Netherlands; 6 Amsterdam Public Health, (Quality of Care), Amsterdam, The Netherlands; 7 Midwifery Academy Amsterdam Groningen, InHolland, Groningen, The Netherlands

**Keywords:** Maternal mental health screening, Depression, Anxiety, PTSD, Migrants, Pregnancy, Postpartum

## Abstract

**Purpose:**

Maternal mental health disorders are prevalent among migrant women. Due to the association of these disorders with adverse pregnancy outcomes, early recognition, and referral are important. This review aims to provide an overview of the literature on mental health screening for migrant women during pregnancy and the postpartum period.

**Methods:**

We systematically searched PubMed, EMBASE, and PsycINFO, covering publications before July 15th, 2024. Database searches were supplemented by a grey literature search, which included a systematic Google and Google Scholar search, hand searching of reference lists, and citation searches. Quantitative, qualitative, and mixed-method studies published in any language were included if they evaluated or validated screening methods for maternal mental health disorders in first-generation migrants. Screening for eligibility, data extraction, and quality appraisal were conducted by two independent researchers. Results were summarized narratively.

**Results:**

Among the 3035 records screened, 30 articles met the inclusion criteria. Our findings indicate that health care providers and migrant women recognize a substantial need for maternal mental health screening, especially for depression, and in a lesser quantity for anxiety and PTSD. We describe a range of barriers and facilitators that impact the quality and feasibility of mental health screening. Research on available screening instruments in migrant populations reports reasonable accuracy, reliability, and validity. However, qualitative evaluations question the screening instruments' cultural appropriateness and translatability.

**Conclusions:**

**T**here is an urgent need for the development and implementation of maternal mental health screening programs tailored to pregnant or postpartum migrants. Further research is essential to enhance the effectiveness and cultural sensitivity of these screening programs.

## Background

The process of migration, especially forced migration, is frequently associated with various stressors that can affect migrants'^1^ mental health (Abubakar et al. 2018; Fair et al. 2020). Before and during a migrant’s journey stressors may include physical violence, gender-based violence, human trafficking, and poor living conditions. Once migrated, people often experience a loss of social networks, low social status, and uncertain asylum procedures (Abubakar et al. 2018; Bustamante et al. 2017; Fair et al. 2020). As a result, the risk of mental health disorders is higher in migrants compared to non-migrants (Close et al. 2016; Fellmeth et al. 2017; Stevenson et al. 2023). Within migrant populations, women more often suffer from mental health disorders compared to men which can partially be attributed to sociocultural roles, psychological attributes, and previous adverse experiences (Hollander et al. 2011; Piccinelli and Wilkinson 2000). Migrants who are pregnant or postpartum are especially vulnerable to mental health disorders, as one in four women experience perinatal depression, one in five perinatal anxiety, and one in 11 perinatal PTSD. (Stevenson et al. 2023).

Mental health disorders during pregnancy are associated with adverse outcomes, including miscarriage, preterm birth, small for gestational age infants, cesarean delivery, and neonatal intensive care unit admittance (Cook et al. 2018; Iliadou et al. 2019; Montagnoli et al. 2020; Nillni et al. 2018). In addition, suicide is a leading cause of maternal death among women with mental health disorders during the postpartum period, accounting for between 9 to 13 percent of all maternal mortality (Howard et al. 2014; Stein et al. 2014). Maternal mental health disorders have also been linked to problems in child development, including insecure attachment, impaired cognitive and social development, and long-term behavioral problems (Gentile 2017; Lima et al. 2018; Tirumalaraju et al. 2020; Zhang et al. 2023).

Early recognition and referral to a specialized health care professional are of great importance to reduce the negative impact of maternal mental health disorders, especially in vulnerable groups such as migrants (Iliadou et al. 2019). A recent review highlighted the lack of attention to psychosocial issues during maternity care, emphasizing that the needs of migrant women extend beyond the physical aspects of pregnancy (Fair et al. 2020). Despite previous studies recommending structured antenatal mental health screening, migrant mothers are not screened to the same extent as non-migrant mothers (Lansakara et al. 2010; Marti-Castaner et al. 2022; Massoudi et al. 2007). This might be because of the challenges health care providers face in screening migrant women, or the barriers women face to access this screening (Fritz and McGregor 2013; Fuggle et al. 2002; Nithianandan et al. 2016; Skoog et al. 2017; Soldati et al. 2021; Willey et al. 2020a). Disparities in mental health screening are of concern, as screening improves the detection rate of mental health disorders in migrant populations and leads to more referrals to a mental health care professional (Willey 2020).

Health care providers use various instruments to screen pregnant migrants for mental health disorders, such as the Edinburgh Postnatal Depression Scale (EPDS), the General Health Questionnaire (GHQ), and the more recently developed Refugee Health Screener 15 (RHS-15) (Cox et al. 1987; Goldberg and Williams 1988; Hollifield et al. 2013). Although these instruments are available in multiple languages, they are not always transculturally validated in migrants. Moreover, the cultural appropriateness of some of the instruments has been questioned in previous studies (Davidson et al. 2010; Playfair et al. 2017). Other difficulties with maternal mental health screening in migrant mothers encompass issues with instrument translations, working with interpreters, low literacy, and the presence of family members (Playfair et al. 2017).

In 2017, Playfair et al. published a scoping review on the identification of antepartum and postpartum mental health disorders among migrant women, which encompassed 13 studies published before 2015. One of the author’s main conclusions was the lack of literature on this topic, which indicated the need for further research (Playfair et al. 2017). As research on migrants increased substantially over the past years, this systematic review aims to provide a current overview of the literature on antenatal and postpartum mental health screening in migrants by answering the following research questions:

1. What are the perspectives of migrant women and health care providers on antenatal and postpartum mental health screening?
2. Which barriers and facilitators can be identified that complicate or facilitate maternal mental health screening for migrant women and health care providers?
3. Which instruments are available and suitable for antenatal and postpartum mental health screening in migrant populations?

## Methods

### Search strategy and selection criteria

We performed a systematic literature search on electronic databases, including PubMed, EMBASE, and PsycINFO, covering publications before July 15th, 2024. Quantitative studies, qualitative studies, mixed-method studies, and reviews were screened for inclusion. The search strategy was originally developed for PubMed and was adjusted according to the specifications of each database. The search terms (Table 1) were performed in combinations using the Boolean operators “AND” and “OR” (Appendix 1). The review was not prospectively registered in an international database. Throughout the article, the term migrant is used as an umbrella term, covering migrants, refugees, and asylum seekers. In the results section, we adhered to the terminology used in the included studies as much as possible. For an explanation of the definitions of asylum seeker, refugee, and migrant see box 1.

**Table 1:** Search terms

Search term	Asylum seekers	Pregnancy	Mental Health
Mesh	refugees	Pregnancy, Maternal Health Services	Mental disorders, Depression
tiab	Refugee, asylum seeker, migrant, displaced person, displaced people, migrant	Maternal, perinatal, postpartum, pregnant, antenatal, postnatal, postpartum, childbirth	Psychiatric, psychological, mental, post-traumatic, posttraumatic, PTSD, depression, anxiety, psychological distress, psychosocial health, stress disorder, mood, schizophrenia, psychosis, psychotic

**Box 1** Definitions of asylum seeker, refugee, and migrant

**Table:** 

Asylum seeker: ‘An individual who is seeking international protection. In countries with individualized procedures, an asylum-seeker is someone whose claim has not yet been finally decided on by the country in which the claim is submitted. Not every asylum-seeker will ultimately be recognized as a refugee, but every refugee was initially an asylum-seeker.’ (UNHCR. The Global Report 2005)
Refugee: ‘a person who, owing to a well-founded fear of being persecuted for reasons of race, religion, nationality, membership of a particular social group, or political opinion, is outside the country of his nationality and is unable to or unwilling to avail himself of the protection of that country’ (UN General Assembly n.d.)AQ
Migrant: ‘an umbrella term reflecting the common lay understanding of a person who moves away from his or her place of usual residence, whether within a country or across an international border, temporarily or permanently, and for a variety of reasons’ (International Organization for Migration (IOM). 2019)

Database searches were supplemented by a comprehensive grey literature search. This included a systematic search of Google Scholar and Google, as well as hand searching of reference lists and citation searches. Websites of appropriate governmental and non-governmental organizations were also searched for grey literature. Where we identified a systematic review, we separately included the studies described in the review that met our inclusion criteria and noted how many studies had been missed by our search. We used the following inclusion and exclusion criteria to identify relevant studies (Table 2).

**Table 2:** Inclusion and exclusion criteria

Inclusion criteria	Exclusion criteria
Studies which include barriers or facilitators to mental health screening in the antenatal and postpartum period for migrant women and/or health care providers	
Studies that qualitatively evaluate or validate screening methods for mental health disorders	
Studies that include either women from migrant, refugee, or asylum-seeker backgrounds	Studies that include women who are not specifically identified as migrants, refugees, or asylum-seekers but for example describe their populations as ‘bilingual’, ‘vulnerable’, or with ‘social risk factors’
Studies in which the study population mainly consists of first generation migrants (women born outside of the host country)	Studies that focus on second generation migrants or internally displaced persons
Studies that include women who are pregnant or up to one year postpartum at the start of the study	
Studies that include health care providers working with women pregnant or postpartum	
Quantitative, qualitative and mixed methods studies	Opinion pieces, commentaries, and case reports Systematic reviews were excluded but their reference lists were fully screened for inclusion
Written in any language	
Full text available	Abstract, conference presentation, or research protocol only, after contacting researchers who conducted the study

All articles were independently reviewed for inclusion by two reviewers (AV and ES). Articles were screened in two rounds; first, the title and abstract were screened and in the second round, the full texts of articles selected in round 1 were reviewed according to the criteria outlined in Table 2. Discrepancies between reviewers regarding inclusion of studies were resolved by discussion and if necessary, involvement of a third reviewer (EF or IP).

### Data extraction

A structured data-charting form to extract information from included studies was pre-defined by all authors to determine which variables to extract. Data extraction included details about the aim of the study, methods, study sample, migration status of participants, screening instruments, mental health disorder screened for, and a summary of relevant findings. For quantitative studies, variables that were related to the accuracy, reliability, and validity of instruments were extracted. The first two authors independently extracted data from the studies, discussed the results, and continuously updated the data-charting form in an iterative process.

### Appraisal of methodological quality

The first two authors individually performed a critical appraisal of methodological quality using the Joanna Briggs Institute (JBI) checklists for qualitative and quantitative studies, and the Mixed Methods Appraisal Tool (MMAT) for mixed method studies (Hong et al. 2018; Lockwood et al. 2015; Moola et al. 2020). Discrepancies were resolved through discussion between the first two authors until reaching consensus. The articles were classified into three categories based on their JBI checklist score: low, moderate, or high. Articles scoring lower than or equal to one-third of affirmative responses were considered of low quality, those scoring between one-third and two-thirds were classified as moderate quality, and those scoring more than or equal to two-thirds were regarded as high quality. All articles were included regardless of their level of quality to present a complete overview of the existing literature.

### Data synthesis and analysis

Data synthesis and analysis were conducted separately for each research question. To capture the perspectives of healthcare providers and migrant women on antenatal and postpartum mental health screening, the first and second authors identified and summarized all relevant paragraphs from the included studies using a narrative approach. For the second research question, barriers and facilitators to screening were extracted from the results section of all qualitative and mixed-method studies. These were then quantified by counting the number of studies in which each barrier or facilitator was reported. To address the third research question, studies were categorized based on the screening instruments they assessed. Data on sensitivity, specificity, and internal consistency were extracted where available and presented in a table. Additionally, healthcare providers' and migrant women's views on specific instruments were summarized narratively. To report the findings of this systematic review we used the PRISMA 2020 checklist for reporting systematic reviews.

## Results

### Article selection

**Fig. 1:**
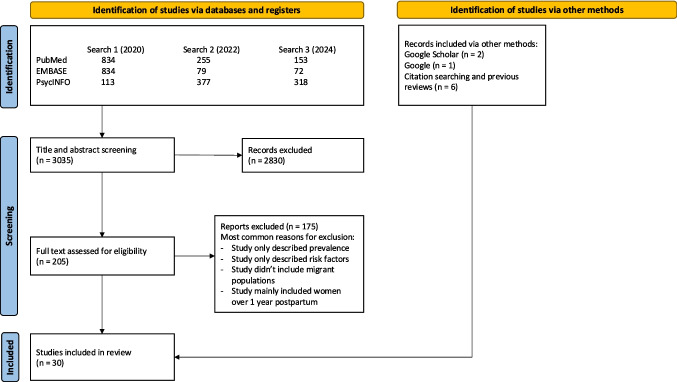
PRISMA flow diagram of inclusion of both initial and update searches

Our initial database search, on November 5th, 2020, identified 1781 studies, of which 19 met the inclusion criteria (see Fig. 1). Six additional studies were identified through reference searching and two through a grey literature search, which was conducted May 2021. Two search updates, took place, the first on November 16th 2022 and the second on July 15th 2024. The first search update identified 711 studies, of which 30 underwent full-text review and four met the inclusion criteria. The second search update resulted in 543 new studies, of which 31 underwent full-text review and zero met the inclusion criteria. The updated grey literature search, on the 30th and 31st of December 2022, identified one additional article, while the grey literature search on July 29th 2024 identified no additional articles. All articles that were eligible for full-text assessment were in English, so there was no need to translate articles to assess their relevance.

### Included studies

Table 3 shows the characteristics of the included studies.

**Table 3:** Characteristics of the included studies

Study characteristics	Number of studies (*n* = 30)
**Study type**	
Quantitative	16
Qualitative	10
Both	4
**Quantitative methods**	
Validation study	8
Survey	4
Screening instrument compared to diagnostic proxy	3
Screening results compared over time	2
Screening results compared between migrant and non-migrant populations	3
**Qualitative methods**	
Interviews	8
Focus group and interviews	3
Focus group	1
Case description	1
Survey	1
**Study country**	
Australia	10
Canada	5
United States of America (USA)	5
Sweden	3
United Kingdom (UK)	2
Thai-Myanmar border	2
Lebanon	1
The Netherlands	1
UK and Bangladesh	1
**Study population**	
Migrant women	21
Health care providers	5
Both	4
**Migrant populations**	
Refugees	8
Asylum seekers	1
Refugees and asylum seekers	4
Refugees and labor migrants	1
Migrant population not specified	11
**Pregnancy status**	
Pregnant	5
Postpartum	15
Both	5
**Mental health disorders**	
Depression	28
Anxiety	9
PTSD	4
Mental health disorders in general	1
**Screening instrument**	
EPDS	20
General Health Questionnaire (GHQ)	4
Refugee Health Screener (RHS)	3
Harvard Trauma Questionnaire (HTQ)	1
Postpartum Depression Screening Scale (PDSS)	1
Center for Epidemiologic Studies Depression Scale (CES-D)	1
Patient Health Questionnaire (PHQ)	1

Most studies focused on one or multiple screening instruments (*n* = 21), while others qualitatively evaluated the screening process in general or concerned barriers and facilitators to mental health screening (*n* = 9). Validation studies compared screening instruments to semi-structured diagnostic interviews such as the Diagnostic Interview Schedule (DIS), the Structured Clinical Interview for DSM diagnosis (SCID), or the Schedule for Affective Disorders and Schizophrenia (SADS) which are considered the gold standard for diagnosis of mental health disorders.

The quality of included studies varied, and the results of the critical appraisal are presented in Table 4 and more elaborately in Appendix 2. Overall, fourteen studies were of high quality, eleven were of moderate quality, and five were of low quality.

**Table 4:** Aims, methods, most relevant findings, and methodological quality of included studies (*n* = 30)

Author Year Country	Study aim	Study method (description of the method used)	Screening instrument (including translation status)	Mental health disorder	Sample	Migration status of participants	Summary of relevant findings	Methodological quality
**Edinburgh Postnatal Depression Scale (EPDS)**								
**Quantitative studies**								
Yoshida et al. 1997 UK	Assess the utility of the Japanese EPDS	At 5 days, 1 month, and 3 months postpartum women completed screening. At 3 months postpartum psychiatric state was assessed with the SADS	EPDS (Japanese translations)	Depression	98 Japanese migrant women	Migrant women, not further specified	The EPDS is not an appropriate screening instrument for depression in this population as there was no cut-off score with an acceptable sensitivity and specificity	Moderate + Psychiatric assessment in duplicate with one psychiatrist blinded to EPDS scores - Follow-up not long enough to assess PPD - Unclear how the screening instrument was translated
Barnett et al. 1999 Australia	Validate translations of the EPDS in Arabic and Vietnamese	Participants completed screening at 6 weeks and 6 months postpartum. Validated against the DIS	EPDS (translated, back-translated, and pilot-tested)	Depression	77 Arabic, 96 Vietnamese women	Mostly first-generation migrant women, not further specified	Best cutoff scores: Vietnamese women: ≥ 15 (sensitivity 100%, specificity 94%) Arabic women: ≥ 10 (sensitivity 78%, specificity 80%)	Moderate + Validated against the gold standard - High loss to follow-up - Statistical analysis not described - Methods not very concise
Small et al. 2007 Australia	Assess whether the EPDS measures the same thing across cultures	Secondary analysis from two national studies: 1. all women who gave birth in a hospital completed the EPDS 6 to 7 months postpartum 2. Migrant women completed the EDPS 6 to 9 months postpartum	EPDS	Depression	318 migrant women from Vietnam, Turkey, and the Philippines, and 1366 women from the general population	First-generation migrants, not further specified	No differences were found in the way women from different cultural and linguistic backgrounds responded to the EPDS	Moderate + Large sample size + Representative populations - EPDS was administered differently in both studies (one by interview other by letter) - No strategy to deal with confounders
Smith 2016 USA	Investigate potential barriers in translating the EPDS to non-English speaking refugee women	Two surveys, one for health care professionals and one for interpreters	EPDS	Depression & anxiety	10 health care providers and 15 interpreters	N/A	Health care providers considered the EPDS less reliable for non-English speaking patients. Interpreters considered the EPDS culturally sensitive, but patient’s education and linguistic differences made translation challenging	Low - Small sample size - Not peer-reviewed - Surveys not validated - Unclear inclusion criteria
Dennis et al. 2016 Canada	Assess the prevalence and persistence of postpartum depressive symptomatology and evaluate the EPDS	Participants completed the EPDS in one of the 13 study languages at 1 and 16 weeks postpartum	EPDS (translated and validated using stringent procedures)	Depression	143 refugees, 369 asylum-seekers, 303 non-refugee migrants, and 310 Canadian-born women	Refugee, asylum seeker, and non-refugee migrant women	EPDS scores at 1 and 16 weeks postpartum were significantly correlated for all subpopulations	Moderate + Elaborate description of methods - High loss to follow-up in the refugee population (31%)
King et al. 2019 Canada	Examine predictors for postpartum depressive symptoms in migrant populations	EPDS scores between 12–14 and 24–28 weeks of gestation and at 1 and 8 weeks postpartum	EPDS (English and French)	Depression	197 Canadian-born women, 60 recent migrants, and 84 less recent migrants	Recent migrants (≤ 5 years stay) and less recent migrants (> 5 years stay)	High EPDS score at 1-week postpartum was a predictor of postpartum depressive symptoms at 8 weeks postpartum for both Canadian-born and migrant women	Moderate - Confounding factors not included in regression analysis
Blackmore et al. 2022b Australia	Validate Dari translation of the EPDS	Participants completed EPDS and SCID during the same prenatal appointment	EPDS (translated, back-translated, and pilot-tested)	Depression & anxiety	52 Dari speaking women	49 refugees and 3 asylum seekers	Good internal consistency. The best cut-off score for depression: ≥ 9 (sensitivity 100%, specificity 88%) The best cut-off score for anxiety: ≥ 5 (sensitivity 100%, specificity 80%)	High + Validated against the gold standard + Blinded (EPDS scores unknown to SCID conductors) - Single center
**Qualitative studies**								
Fritz and McGregor 2013 USA	Describe strategies for PPD screening for women from two migrant populations	Case study: description of two cases	EPDS	Depression	1 Yemen and 1 Punjabi woman	Migrant women, not further specified	Challenges in screening for PPD in these populations and considerations when screening with interpreters	Low - No description of methods
O’Mahony et al. 2013 Canada	Explores what influences the way in which migrant and refugee women seek help for PPD	In-depth interviews with or without an interpreter	EPDS	Depression	8 refugee women and 22 migrant women	Non-European women with a migrant or refugee status living in Canada for < 10 years	Migrant or refugee women might not give honest answers to EPDS questions because they do not fully understand the seriousness of PPD or are fearful of being alienated or disrupting family harmony	High + Diversity of sample - Small sample size - Influence of the researcher on the research, and vice-versa, not addressed
Nithianandan et al. 2016 Australia	Investigate barriers and enablers to implementing maternal mental health screening for women with a refugee background	Semi-structured interview study, with thematic analysis	EPDS	Depression, anxiety & PTSD	28 health care providers, 9 migrant women	Migrant women with a refugee or asylum seeker background	Participants considered maternal screening for depression, anxiety, and PTSD necessary. Barriers and facilitators to implementation were identified	High + Data saturation reached - Influence of the researcher on the research, and vice-versa, not addressed
Skoog et al. 2017 Sweden	Elucidate child health service nurses’ experiences of identifying PPD in non-Swedish-speaking migrant mothers	A semi-structured interview study with an inductive approach	EPDS	Depression	13 child health service nurses who work with migrant mothers	N/A	Health care providers considered screening for PPD important and explained the challenges they encounter while screening	High + Clear and elaborately described methodology - Unclear whether saturation was reached
Skoog et al. 2019 Sweden	Elucidate non-native-speaking migrant mothers’ experiences of PPD screening	a semi-structured interview study with an inductive approach	EPDS	Depression	13 women with a refugee background postpartum	Twelve refugees with a residence permit and one asylum seeker	Participants considered PPD screening important and describe barriers to talking about mental health	High + Clear and elaborately described methodology - Small sample size
Yu et al. 2019 USA	Develop and evaluate a survey about migrant women’s experiences with PPD screening	After survey development, experts rated content validity and migrant women provided feedback	EPDS	Depression	9 experts and 12 Chinese women	Migrant women, not further specified	Participants considered PPD screening important, did not consider family a barrier and questioned the linguistic appropriateness of the EPDS	Moderate - Sample not representative of the target population - influence of the researcher on the research, and vice-versa, not addressed
Willey et al. 2020a Australia	Determine whether a digital maternal mental health screening program is feasible and acceptable for migrant women	One focus group and 13 semi-structured telephone interviews. Inductive and deductive approach	EPDS	Depression & anxiety	22 women, 1 antenatal and 21 postpartum	17 migrant women with a refugee background and 5 with a migrant background	Women consider maternal screening for depression and anxiety important and acceptable. Barriers and facilitators to mental health screening were discussed	High + Multiple sources of data - Selection bias - Telephone interviews instead of face to face
**Mixed methods studies**								
Stapleton et al. 2013 Australia	Explore the utility of the EPDS in refugee women	Mixed methods with multiple data sources, including 4158 hospital records, 190 chart audits, 189 surveys, 3 interviews, and 8 focus groups	EPDS	Depression	Health care providers refugee women, community stakeholders, and research assistants	Women with a refugee background	The appropriateness of the EPDS is questionable because of linguistic and cultural issues	Moderate + wide variety of data sources - unclear who conducted the qualitative analysis - Unclear which data originates from which source and no description of survey analysis
Ing et al. 2017 Thai-Myanmar border	To validate the Burmese and Karen versions of the EPDS and to assess its acceptability	Participants completed the EPDS and afterward the SCID between 4 and 16 weeks postpartum. After recruitment, members of the study team participated in a focus group	EPDS	Depression	675 Karen or Burmese women	Refugee and migrant women	EPDS showed high accuracy and reasonable internal consistency. However, acceptability to local staff was low because the EPDS was considered difficult to accurately translate and migrant women found it hard to complete	Moderate + SCID interviewers were blinded to the results of the EPDS - Number and background of focus group attendants unclear - Qualitative analysis not described
Willey et al. 2020b Australia	Evaluate the acceptability and feasibility of maternal mental health screening for refugee women from the health care provider's perspective	Mixed methods design guided by the Normalization Process Theory. Multiple data sources including an online survey, focus groups, and semi-structured interviews with professionals	EPDS	Depression & anxiety	31 health care professionals	-	Participants valued a newly introduced maternal mental health screening program. Professionals discussed multiple barriers and facilitators to screening	High + mixed methods approach - Single center study, medical staff not included
**General Health Questionnaire GHQ**								
**Quantitative studies**								
Watson and Evans 1986 UK	Assess if PPD can be measured across cultures	Interview assessment, self-assessment, and the GHQ at 8 weeks, 8 months, and 14 months postpartum	GHQ (translated and back-translated)	Depression	28 Bengali migrant, 24 non-Bengali migrant, and 49 non-migrant mothers	Migrant women, not further specified	Women from different cultures responded similarly to PPD assessment with the GHQ	Low + Random selection - Small sample - Translated GHQ not validated or pilot tested
Yeung and Schwartz 1986 USA	Examine the level of psychiatric morbidity in Chinese obstetrical patients	During their initial obstetric visit, women completed the GHQ-28 and SCID	GHQ-28 (translated)	All mental health disorders	124 Asian migrant women	Migrant women, not further specified	The GHQ was useful while screening for psychiatric morbidity. Sensitivity of 74% and specificity of 98% with a cutoff score of ≥ 9	Moderate + Blinded (SCID assessor blinded to GHQ outcome) - No timeframe - Translated instrument not validated or pilot tested
**Refugee Health Screener (RHS)**								
**Quantitative studies**								
Fellmeth et al. 2018 Thai Myanmar border	Determine the validity and acceptability of Sgaw Karen and Burmese RHS-15	During the first trimester of pregnancy, the RHS-15 and SCID were administered verbally	RHS-15 (translations were obtained from RHS-15 authors)	Depression	235 Burmese-speaking and 275 Sgaw Karen-speaking women	Labor migrants (57,1%) and refugees (42,9%)	Best cutoff scores: Burmese: ≥ 14 (sensitivity 82%, specificity 76%) Sgaw Karen: ≥ 15 (sensitivity 88%, specificity 81%)	High - Not blinded (SCID and RHS-15 by the same person) - SCID not conducted by psychiatrists
Alnaji et al. 2021 Lebanon	Validate Arabic translation of the RHS-13	Participants completed screening within 1 year postpartum by phone interview. Validated against PHQ-9, GAD-7 and PC_PTSD5	RHS-13 (Arabic)	Depression, anxiety & PTSD	103 Syrian refugee women postpartum	Refugees	RHS-13 correlates well with PHQ-9 and GAD-7. Weak correlation with PC_PTSD5. Good internal consistency. Best cutoff score: ≥ 12	Low - Insufficient description of methods - Not validated against the gold standard - Verbal administration of screening over the phone
**Qualitative studies**								
Soldati et al. 2021 Netherlands	Evaluate asylum-seeking women's perception of prenatal mental health screening and the RHS-15	a semi-structured interview study with an inductive approach	RHS-15	Depression, anxiety, and PTSD	8 pregnant asylum seekers	Asylum seekers	Main themes: 'Importance of mental health screening', 'Talking about mental health', and 'Use of the RHS-15'	High + Topic list developed by a multicultural team - Convenience sampling of participants
**Other or multiple instruments**								
**Quantitative studies**								
Fuggle et al. 2002 UK and Bangladesh	Determine the utility of a translated Bengali version of the EPDS	Participants self-completed the EPDS and GHQ between 8 and 12 weeks (in Bangladesh) or between 8 weeks and 12 months postpartum (in London)	EDPS and GHQ-28 (Translated and back-translated)	Depression	48 Bangladeshi women, 22 in Bangladesh and 26 in London	In London: Second- and first-generation migrant women, not further specified	EPDS had a positive association with GHQ. EPDS showed adequate internal consistency (alpha = 0.73). Translation of EPDS and GHQ to Bengali posed a challenge	Moderate - Selection bias - Selection period and inclusion criteria unclear
Le et al. 2010 USA	Examine psychometric properties of the Spanish PDSS	Participants were part of a preventive intervention trial. The screening was conducted between 6 and 8 weeks postpartum	PDSS (Spanish)	Depression & anxiety	155 Latina migrants at risk for depression	Migrant women, not further specified	Spanish PDSS had an excellent internal consistency (Cronbach’s α: 0.97) and was positively correlated with the BDI-II. Internal consistency for the anxiety subscale was moderate (Cronbach’s α: 0.72)	High + Exposure measured in a valid and reliable way - No strategies to deal with confounders
van Lieshout et al. 2011 Canada	Assess measurement invariance of the CES-D across migrant and non-migrant women	Screening at 2 months postpartum	CES-D (English)	Depression	215 English-speaking migrant women and 441 non-migrant women	First generation Migrants with a duration of stay < 10 years or women who identified themselves as refugees	Previously tested models of the factor structure of the CES-D performed poorly in the migrant group. A novel 4-factor structure was introduced, and a 15-item version was established that demonstrated measurement invariance across migrant and non-migrant groups	High - Selection bias (only English-speaking migrant women)
Skoog et al. 2022 Sweden	Test the feasibility of an educational intervention for screening migrant mothers for PPD	Pretest–posttest experimental design measured participants' acceptability of the intervention	None	Depression	30 Child health service nurses	N/A	The intervention was feasible and improved participants' self-estimated cultural competence in screening for PPD	High + Methodology elaborately described - Questionnaires adjusted without validation
Blackmore et al. 2022a Australia	Investigate screening properties of the HTQ for PTSD	Participants completed HTQ and trauma module from the SCID during the same prenatal appointment	HTQ (verbally translated into Dari)	PTSD	52 Dari speaking women	49 refugees and 3 asylum seekers	The best cut-off score for PTSD screening: ≥ 2.25 (sensitivity 100%, specificity 76%, PPV 20%)	High + Validated against the gold standard + Blinded (HTQ scores unknown to SCID conductors) - Single center
**Qualitative studies**								
Teng et al. 2007 Canada	Explore health care worker’s experiences of providing care to recent migrant women who suffer from PPD	Semi-structured interviews analyzed according to the grounded theory approach	None	Depression	16 health care providers, of which seven migrants	N/A	Health care providers considered existing instruments inadequate for PPD screening in migrant women	High - Philosophical perspective unclear (influence of the researcher on the research, and vice-versa, not described)
Kamara 2019 Australia	Implementation and evaluation of screening for PPD with the EPDS	Refugee women completed the EPDS in the first year postpartum. Health care providers were surveyed about their experiences before and after an education session on the EPDS	EPDS and PHQ-2 (English with interpreters if necessary)	Depression	10 refugee women, 4 health care providers	Refugee women	The EPDS was more sensitive to mild symptoms of depression compared to the PHQ-2. Health care providers preferred the EPDS over the PHQ-2	Low - Very small sample - Mismatch between aim and methods (objectives too elaborate for questionnaires) - Asking health care providers about their opinion on the EPDS just after training about its advantages
**Mixed methods studies**								
Matthey et al. 1997 Australia	Evaluate the diagnostic interview schedule to diagnose PPD in Arabic and Vietnamese women	Women completed screening and the DIS at 6 weeks and 6 months postpartum. After the 6-month assessment, a small sample of women was interviewed	EPDS, GHQ-30	Depression & anxiety	126 Vietnamese, 125 Arabic, and 128 Anglo-Celtic women	Migrant women, not further specified	Participants preferred the EPDS and GHQ-30 over the DIS. Women considered questions in screening instruments culturally inappropriate	Moderate + Quantitative and qualitative data - Unclear qualitative research question and analysis

In the description of the methodological quality of studies, the symbol " + " denotes a strength, while the symbol "-" represents a limitation

### Health care providers and migrant women’s views on antenatal and postpartum mental health screening

In multiple studies, both health care providers and migrant women perceived a need for antenatal and postpartum mental health screening for depression, anxiety, and PTSD, and acknowledged the benefits of early detection and referral (Nithianandan et al. 2016; Skoog et al. 2017; Soldati et al. 2021; Willey et al. 2020a; Willey et al. 2020b; Yu et al. 2019). In qualitative studies, the most common reason why migrant women considered mental health screening important was that women would not initiate a conversation about mental health with their health care providers (O’Mahony et al. 2013; Soldati et al. 2021; Willey et al. 2020a). Migrant women also described how mental health screening made them feel supported as they valued being able to express their feelings to their health care providers (Nithianandan et al. 2016; Skoog et al. 2019; Soldati et al. 2021; Willey et al. 2020a). Women even seem to appreciate screening for PPD when the concept of PPD and the purpose of the screening is unclear to them (Skoog et al. 2019; Yu et al. 2019). The implementation of a mental health screening program helped health care providers identify issues that would have previously gone undetected (Willey et al. 2020b).

### Barriers and facilitators to antenatal and postpartum mental health screening

**Fig. 2:**
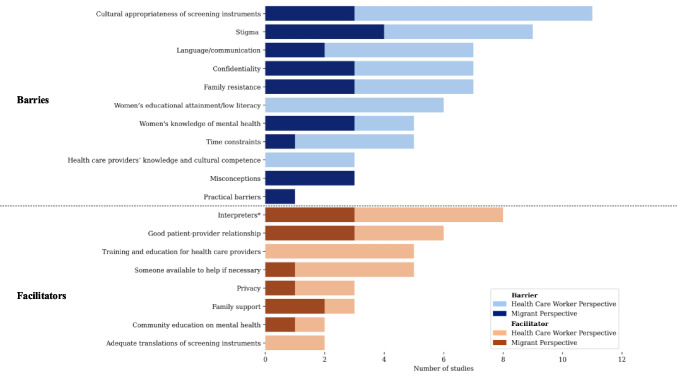
Barriers and facilitators to antenatal and postpartum mental health screening for migrants. * Although interpreters were considered to facilitate mental health screening, many studies describe considerations and challenges in working with interpreters (see Appendix 3)

The most common barriers to adequate mental health screening mentioned in studies included the cultural appropriateness of screening instruments, stigma, language/communication, confidentiality, and family (see Fig. 2). The most common facilitators were interpreters, a good patient-provider relationship, training/education for health care providers, and the availability of someone to assist women when they complete the screening. For an elaborate description of the barriers and facilitators see Appendix 3.

### Screening tools

Table 5 presents the sensitivity, specificity, and internal consistency (as a determinant for reliability) of various screening tools as reported in individual studies. The ensuing paragraphs provide a comprehensive description of the evidence per screening instrument, summarizing both quantitative and qualitative data.

**Table 5:** Sensitivity, specificity, and internal consistency of screening instruments

Study	Screening Instrument	Language	Mental health disorder	Recommended cutoff ( ≥)	Sensitivity (%)	Specificity (%)	Internal consistency (Cronbach’s alpha)
**EPDS**							
Yoshida 1996	EPDS	Japanese	PPD	none			
Barnett 1999	EPDS	Vietnamese	PPD	15	100	94	
		Arabic		10	78	80	
Fuggle 2002	EPDS	Bengali	PPD				0.73
Small 2007	EPDS	Vietnamese, Turkish, and Filipino	PPD				≥ 0.80
Ing 2017	EPDS	Karen	PPD	10	100	99	0.59
		Burmese		10	100	97	0.82
Blackmore 2022	EPDS	Dari	PPD	9	100	88	0.79
			Anxiety	5	100	80	
**GHQ**							
Yeung 1986	GHQ	Chinese	DSM-III diagnosis	9	74	98	
**RHS**							
Fellmeth 2018	RHS-15	Burmese	PPD	14	82	76	0.63
		Karen		15	88	81	0.56
Alnaji 2021	RHS-13	Arabic	Depression	25*	81*	9*	0.80
			Anxiety	25*	100*	14*	
			PTSD	25*	32*	15*	
**HTQ**							
Blackmore 2022	HTQ	Dari	PTSD	2.25	100	76	
**PDSS**							
Le 2010	PDSS	Spanish	Depression				0.97
			Anxiety				0.72

* Validated against diagnostic proxies and not the gold standard

#### EPDS

The EPDS showed good sensitivity and specificity in screening for both depression and anxiety in almost all studies, while the reliability, measured by internal consistency, was moderate to good (Table 5). Yoshida et al. however found that the EPDS did not determine depression risk in Japanese women, as there was no cut-off score with an acceptable balance between sensitivity and specificity (Yoshida et al. 1997). Dennis et al. and King et al. showed good predictive validity of the EPDS for depression, as women’s EPDS score at one-week postpartum was a predictor for persistent depressive symptoms at 8 and 16 weeks after childbirth (Dennis et al. 2016; King et al. 2019). Small et al. reported good construct validity (significant inter-item correlation for all questions) and internal reliability (Cronbach’s α ≥ 0.8) of the EPDS for depression within migrant and non-migrant populations (Small et al. 2007). Recommended cut-off scores for depressive symptomatology varied between 9 and 15, while one study suggested a cut-off score of 5 for the anxiety sub-scale.

Qualitative data show mixed results for the EPDS. Migrant women generally perceive the EPDS as an appropriate screening instrument, while health care providers question its appropriateness but consider it the best instrument available for postpartum depression screening (Nithianandan et al. 2016). For example, in two studies, both Vietnamese and Arabic migrant women, Vietnamese research assistants, and midwives preferred the EPDS over other screening instruments (GHQ and PHQ) (Kamara 2019; Matthey et al. 1997). Health care providers mostly question the EPDS’s cultural appropriateness and express concerns about the accuracy of translations (Fuggle et al. 2002; Ing et al. 2017; Nithianandan et al. 2016; Skoog et al. 2017; Smith 2016). Both health care providers and migrant women raise concerns about the translatability and cultural appropriateness of certain questions, such as Q6 and Q10, and the use of a 4-point Likert scale (Blackmore et al. 2022b; Fuggle et al. 2002; Ing et al. 2017; Matthey et al. 1997; Skoog et al. 2019; Skoog et al. 2017; Smith 2016; Stapleton et al. 2013; Willey et al. 2020b; Yu et al. 2019). In the study by Ing et al. for example, acceptability of the EPDS among local staff was low because staff felt it was inappropriate to use an instrument women found so difficult to complete. Additionally, Ing et al. described issues in accurately conveying the meaning of questions in Karen and Burmese (Ing et al. 2017).

#### GHQ

In one study, the GHQ had favorable sensitivity and specificity for detecting any DSM-III diagnosis in pregnant Chinese migrants (Table 5) (Yeung and Schwartz 1986). Watson and Evans compared the assessment of PPD symptoms with the GHQ, self-assessment, and interviewer's assessment between Bengali-speaking migrant, English-speaking migrant, and non-migrant mothers. The study revealed agreement among all three instruments and found similar GHQ scores across groups. This indicates that mothers from diverse cultural backgrounds respond similarly to PPD assessment with the GHQ (Watson and Evans 1986). Fuggle et al. showed a modest correlation between the GHQ and EPDS scores (Pearson’s correlation coefficient r = 0.422, *p* < 0.003) (Fuggle et al. 2002).

Qualitative results from Matthey et al. indicate that both Vietnamese and Arabic women have concerns about the cultural appropriateness of certain GHQ questions, while English-speaking women have no issues with any of the items (Matthey et al. 1997). Fuggle et al. showed that translating the GHQ into Bengali posed various challenges and was less successful than translating the EPDS, as more items in the GHQ lost their initial meaning in back-translation (Fuggle et al. 2002).

#### RHS

Fellmeth et al. showed reasonable sensitivity and specificity for the RHS-15 when screening for depression in both Burmese and Karen translations and moderate internal consistency (Table 5) (Fellmeth et al. 2018). On the other hand, Alnaji et al. found high sensitivities but low specificities for the RHS-13 when compared to diagnostic proxies for depression, anxiety, and PTSD (Alnaji et al. 2021). In the study by Fellmeth et al. 44% of all women screened positive on the RHS-15 with optimal cutoff scores, determined by the best balance between sensitivity and specificity (Fellmeth et al. 2018).

Although qualitative data on the RHS were limited, Soldati et. al. reported that asylum-seeking women considered the RHS-15 acceptable, while Fellmeth et al. described that health care providers preferred the SCID (gold standard) as it often took less time to administer (Fellmeth et al. 2018; Soldati et al. 2021).

#### Other screening instruments

Le et al. demonstrated excellent internal consistencies of the PDSS and showed a positive correlation with the BDI-II in a sample of Spanish-speaking migrants (Le et al. 2010). Lieshout et al. assessed the measurement invariance of the CES-D between migrant and non-migrant women. Authors found that previously tested models of the factor structure of the CES-D performed poorly in their migrant group. They proposed a new factor structure that demonstrated measurement invariance across migrant and non-migrant groups (Lieshout et al. 2011). Blackmore et al. validated the screening properties of the HTQ for PTSD against the SCID-V. The authors recommended a cutoff score of ≥ 2.25, which demonstrated good sensitivity (100%) and specificity (76%) in detecting PTSD (Blackmore et al. 2022a).

No studies included qualitative outcomes on either the PDSS, BDI-II, CES-D, or HTQ specifically.

## Discussion

This review aimed to provide an overview of existing literature on mental health screening for migrant women during pregnancy and the postpartum period. In all studies, both health care providers and migrant women express a substantial need for maternal mental health screening, especially for depression, and in a lesser quantity for anxiety and PTSD. Most studies examining barriers and facilitators to screening were qualitative and revealed a range of factors that impact the quality and feasibility of mental health screening. Research on available screening instruments in migrant populations, although limited, report reasonable accuracy, reliability, and validity. Qualitative evaluations however are often less positive, with health care providers and migrant women often questioning screening instrument’s cultural appropriateness and translatability.

This review highlights the importance of mental health screening for migrant populations, especially refugees and asylum seekers (Magwood et al. 2022; Wickberg et al. 2020). Screening during pregnancy and the postpartum period for these populations is important as mental health disorders are common and the effects of poor mental health on women and their babies can be detrimental (Cook et al. 2018; Fellmeth et al. 2017; Field 2010, 2011; Gentile 2017; Howard et al. 2014; Iliadou et al. 2019; Lindahl et al. 2005; Montagnoli et al. 2020; Nillni et al. 2018; Stein et al. 2014). Extensive research has reported that women from diverse cultural and refugee backgrounds do not proactively seek help for mental health disorders, despite a desire to discuss such concerns with their health care providers (Chan et al. 2002; Dennis and Chung-Lee 2006; Nithianandan et al. 2016; Soldati et al. 2021; Teng et al. 2007). Programs that combine maternal mental health screening with enhanced support have proven to be both clinically valuable and cost-effective and therefore have the potential to improve perinatal outcomes in high-risk populations, such as migrant women (Howard et al. 2014; Iliadou et al. 2019; Magwood et al. 2022; O’Connor et al. 2016; Willey 2020). Therefore, national screening programs for pregnant migrant women should be implemented in all host countries. Furthermore, user-friendly referral pathways should be established to enable health care providers to direct clients to appropriate services following a positive screen. To encourage migrant women and communities to discuss mental health concerns with their health care provider, a national campaign in multiple languages through various channels, can be an effective approach (Booth et al. 2018; Freeman et al. 2015).

To optimize the effectiveness of maternal mental health screening programs, they must be designed to overcome barriers and maximize the utilization of facilitators. Most barriers and facilitators in this review are comparable to those found in maternity care for migrant populations in general, or identified in studies regarding mental health interventions for other populations (Heslehurst et al. 2018; Le et al. 2022; Lopez et al. 2018). These barriers and facilitators inform recommendations regarding the optimal approach to maternal mental health screening in migrant populations. To optimize screening procedures women should be enabled to complete screening in private, preferably before antenatal care appointments so they can discuss outcomes with their health care provider afterward (Fritz and McGregor 2013; Magwood et al. 2022; Nithianandan et al. 2016; Willey et al. 2020b). To address struggles with independently filling in screening instruments, a possible solution could be to develop and validate audio versions of instruments, possibly even with built-in answers to common questions (Willey et al. 2020a). Furthermore, screening results should preferably be discussed with a health care provider that cares for a woman over a longer period and can therefore build a trusting relationship (Nithianandan et al. 2016; Soldati et al. 2021). During pregnancy, this is typically a midwife or doctor, while after childbirth, child health care nurses or midwives might be the most appropriate options (Nithianandan et al. 2016; Soldati et al. 2021). Regardless of who discusses screening results with women, various studies highlight the importance of an official interpreter present to overcome language barriers as it is easier for women to discuss mental health in their native language (Donnelly and Leavey 2022; Magwood et al. 2022). Further research should compare different screening methods and quantify barriers and facilitators to aid the development and culturally appropriate implementation of evidence-based mental health screening guidelines.

There is currently no consensus in international literature regarding the optimal screening instrument for mental health disorders in migrant women during pregnancy and after childbirth. The reasonable accuracies, validities, and reliabilities described in this review suggest that screening instruments could have some utility in the assessment of mental health symptoms in migrant groups. However, the negative qualitative evaluations and considerable diversity in cut-off scores between women from different cultural backgrounds may complicate the utility of screening instruments. This raises a fundamental question of whether it is possible to compare mental health symptoms across cultures due to linguistic and cultural differences. To answer this, further research should investigate which instrument is most suitable to screen for maternal mental health disorders in migrant populations by comparing the accuracy, validity, and reliability of instruments, as well as qualitatively exploring their utility and suitability according to women and health care providers. In addition, further research should prioritize maternal anxiety and PTSD screening for migrant women, as this review shows a notable lack of literature on these disorders specifically. All further research should consider the diversity of the migrant population and should prioritize instruments with the capacity to screen for multiple mental health disorders. In the meantime, the implementation of screening programs is of paramount importance and cannot be delayed while waiting for further research. Until further research reaches a consensus regarding the optimal screening test, health care providers might use the screening test recommended by their current guidelines.

### Strengths and limitations

The main limitations of this review were the limited number of relevant studies and the considerable heterogeneity of study designs, migrant populations, and outcomes. Due to the limited number of studies available we were only able to evaluate mental health screening methods for the migrant population as a whole instead of recognizing the different subgroups within this population, while different groups might have different health needs. This hindered our ability to compare screening instruments quantitatively. An important strength was the comprehensive and rigorous search strategy, which had no language restriction and was developed in collaboration with researchers in the field and a skilled librarian. Furthermore, the inclusion of both quantitative and qualitative studies provides a comprehensive understanding of the topic and enhances the robustness of our findings. This review highlights significant gaps in existing evidence and offers suggestions for policy development and implementation. Additionally, we provide actionable insights for health care providers who offer maternity care to migrant women.

## Conclusion

This systematic review highlights the urgent need for the development and implementation of maternal mental health screening programs tailored to pregnant or postpartum migrant populations. Given the limited literature currently available, further research is essential to enhance the effectiveness and cultural sensitivity of these screening programs. Addressing these gaps is critical to ensure inclusive care for migrant women and their children, ultimately advancing equity in maternal and child health care.

## Figures and Tables

**Fig. 3:**
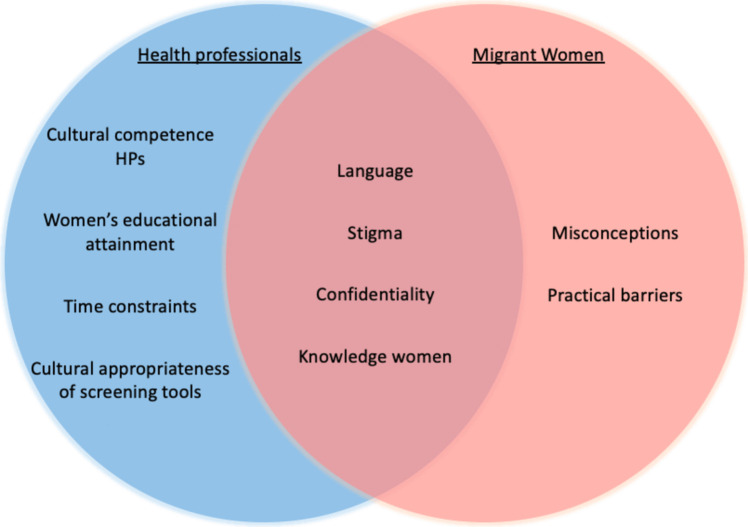
Barriers identified by migrant women and health care providers in included studies

**Fig. 4:**
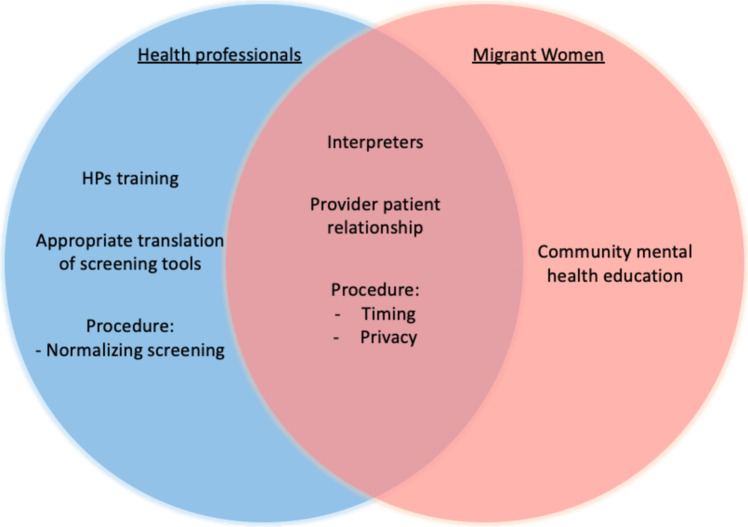
Facilitators identified by migrant women and health care providers in included studies

## Data Availability

The structured data-charting form we used to extract information from included studies and the data extracted from included studies are published as supplementary material.

## Appendix 1 Search strategy per database

### PubMed

("refugees"[MeSH] OR refugee*[tiab] OR asylum seeker[tiab] OR asylum seekers[tiab] OR migrant*[tiab] OR displaced person*[tiab] OR displaced people[tiab] OR immigrant[tiab] OR immigrants[tiab]) AND ("pregnancy"[MeSH] OR "Maternal Health Services"[Mesh] OR maternal[tiab] OR perinatal[tiab] OR postpartum[tiab] OR pregnant*[tiab] OR antenatal[tiab] OR postnatal[tiab] OR post-partum[tiab] OR childbirth[tiab]) AND ("mental disorders"[MeSH] OR "Depression"[Mesh] OR psychiatric[tiab] OR psychological[tiab] OR mental[tiab] OR post-traumatic [tiab] OR posttraumatic[tiab] OR PTSD[tiab] OR depressed[tiab] OR depression[tiab] OR anxiety[tiab] OR psychological distress*[tiab] OR psychosocial health[tiab] OR stress disorder*[tiab] OR mood[tiab] OR schizophren*[tiab] OR psychosis[tiab] OR psychotic[tiab])

### Embase

('migrant'/exp OR 'migrant' OR 'refugee'/exp OR 'refugee' OR 'asylum seeker'/exp OR 'asylum seeker' OR 'refugee camp'/exp OR 'refugee camp' OR 'immigrants'/exp OR 'immigrant') AND ('pregnancy'/exp OR 'maternal health service':ti,ab,kw OR 'maternal care':ti,ab,kw OR 'perinatal care':ti,ab,kw OR 'childbirth':ti,ab,kw OR 'prenatal care':ti,ab,kw OR 'postnatal care':ti,ab,kw) AND ('mental diseases'/exp OR 'depression'/exp OR 'mental disease':ti,ab,kw OR 'posttraumatic stress disorder':ti,ab,kw OR 'depression':ti,ab,kw OR 'anxiety disorder':ti,ab,kw OR 'psychosis':ti,ab,kw OR 'schizophrenia':ti,ab,kw OR 'psychosocial health':ti,ab,kw)

### PsycINFO

(migrant or refugee or asylum seeker or displaced person or displaced people or immigrant) AND ( pregnancy or pregnant or prenatal or antenatal or perinatal or maternal or postnatal or postpartum or birth or mother or maternal or postnatal or childbirth) AND ( psychiatric or mental health or mental illness or mental disorder or psychiatric illness or psychological or posttraumatic or PTSD or depression or depressed or anxiety or psychological health or psychosis or psychotic or schizophrenia or mood or stress disorder)

### Grey literature search

#### Google

For each screening test and NGO, we searched the first 20 pages. We used the following two search terms:

1. Pregnant ''*Screening test*'' ''asylum seeker'' OR refugee OR migrant filetype:pdf
2. ‘’*NGOs*’’ Pregnant ''mental health screening'' ''asylum seeker'' OR refugee OR migrant filetype:pdf
  1. Screening tests searched: GHQ-12, R4U, SRQ, Mind2Care, RHS-15, Harvard Trauma Questionnaire, EPDS, mental health screening.
  2. NGOs searched: WHO, UNHCR, OMI, OXFAM, and Save the Children.

#### Google scholar search

For each screening test and NGO, we searched the first 20 pages. We used the following two search terms:

1. Pregnant *Screening test* screening ''asylum seeker'' OR refugee OR migrant
2. *NGO* pregnant ''mental health screening'' ''asylum seeker'' OR refugee OR migrant
  1. Screening tests searched: GHQ-12, R4U, SRQ, Mind2Care, RHS-15, Harvard Trauma Questionnaire, EPDS, mental health screening.
  2. NGOs searched: WHO, UNHCR, OMI, OXFAM, and Save the Children.

#### Scanning reference lists

We scanned reference links of all included articles and related reviews not included in the study for additional articles and grey literature.

#### Contacting experts

We contacted the authors of included studies for which only a conference abstract was available online by e-mail. We found 1 conference abstract eligible for inclusion which concerned one of our studies.

## Appendix 2

Table 6

**Table 6:** Critical appraisal of methodological quality per study

Study	JBI checklist	Quality	Total Yes	Q1	Q2	Q3	Q4	Q5	Q6	Q7	Q8	Q9	Q10	Q11
Barnett et al. (1999)	Cohort	Moderate	5	Unclear	Yes	Yes	Unclear	No	Yes	Yes	Yes	No	No	Unclear
Dennis et al. (2016)	Cohort	Moderate	7	Yes	Yes	Yes	No	No	Yes	Yes	Yes	No	Unclear	Yes
King et al. (2019)	Cohort	Moderate	7	Yes	Yes	Yes	Yes	No	Yes	No	No	Yes	No	Yes
Skoog et al. (2022)	Cohort	High	8	N/A	N/A	Yes	Yes	Yes	Yes	No	Yes	Yes	Yes	Yes
Yoshida et al. (1997)	Cohort	Moderate	5	Yes	Yes	Unclear	Yes	No	Yes	Yes	No	No	No	Unclear
Watson and Evans (1986)	Cohort	Low	3	No	N/A	N/A	No	No	Unclear	No	Yes	Yes	No	Yes
van Lieshout et al. (2011)	Cross-sectional	High	7	Yes	Yes	No	Yes	Yes	Yes	Yes	Yes			
Blackmore et al. (2022b)	Cross-sectional	High	6	Yes	Yes	Yes	Yes	N/A	N/A	Yes	Yes			
Small et al. (2007)	Cross-sectional	Moderate	3	Unclear	Yes	Yes	Unclear	No	No	No	Yes			
Smith (2016)	Cross-sectional	Low	0	No	No	N/A	N/A	No	No	No	Unclear			
Fuggle et al. (2002)	Cross-sectional	Moderate	3	Unclear	No	Yes	Unclear	No	No	Yes	Yes			
Yeung and Schwartz (1986)	Cross-sectional	Moderate	4	Yes	No	Yes	Yes	No	No	Yes	Unclear			
Blackmore et al. (2022a)	Cross-sectional	High	6	Yes	Yes	Yes	Yes	N/A	N/A	Yes	Yes			
Le et al. (2010)	Cross-sectional	High	7	Yes	Yes	Yes	Yes	Yes	No	Yes	Yes			
Alnaji et al. (2021)	Cross-sectional	Low	2	No	No	No	Yes	Unclear	Unclear	No	Yes			
Fellmeth et al. (2018)	Cross-sectional	High	4	Yes	Yes	N/A	Yes	N/A	N/A	No	Yes			
Fritz and McGregor (2013)	Qualitative	Low	0	Unclear	Unclear	Unclear	Unclear	No	No	No	Unclear	No	Unclear	
Nithianandan et al. (2016)	Qualitative	High	7	Unclear	Yes	Yes	Yes	Yes	No	No	Yes	Yes	Yes	
O’Mahony et al. (2013)	Qualitative	High	8	Yes	Yes	Yes	Yes	Yes	No	No	Yes	Yes	Yes	
Skoog et al. (2017)	Qualitative	High	10	Yes	Yes	Yes	Yes	Yes	Yes	Yes	Yes	Yes	Yes	
Skoog et al. (2019)	Qualitative	High	9	Yes	Yes	Yes	Yes	Yes	No	Yes	Yes	Yes	Yes	
Willey et al. (2020a)	Qualitative	High	7	Yes	Yes	Unclear	Yes	Yes	No	No	Yes	Yes	Yes	
Yu et al. (2019)	Qualitative	Moderate	6	Yes	Yes	Unclear	Yes	Yes	No	No	Yes	Unclear	Yes	
Kamara (2019)	Qualitative	Low	3	Unclear	Yes	No	No	No	Yes	No	Unclear	Yes	No	
Teng et al. (2007)	Qualitative	High	7	Unclear	Yes	Yes	Yes	Yes	No	No	Yes	Yes	Yes	
Soldati et al. (2021)	Qualitative	High	9	No	Yes	Yes	Yes	Yes	Yes	Yes	Yes	Yes	Yes	
Ing et al. (2017)	Cross-sectional	Moderate	5	Yes	Unclear	Yes	Yes	N/A	N/A	Yes	Yes			
	Qualitative		4	Unclear	Unclear	Yes	No	Yes	No	No	No	Yes	Yes	
Stapleton et al. (2013)	Cross-sectional	Moderate	3	No	No	Yes	N/A	Yes	No	Unclear	Yes			
	Qualitative		5	Yes	Yes	No	No	Yes	No	No	No	Yes	Yes	
	MMAT		2	Yes	Yes	No	No	No						
Willey et al. (2020b)	Cross-sectional	High	4	Yes	Yes	N/A	N/A	N/A	N/A	Yes	Yes			
	Qualitative		8	Yes	Yes	Yes	Yes	Yes	No	No	Yes	Yes	Yes	
	MMAT		5	Yes	Yes	Yes	Yes	Yes						
Matthey et al. (1997)	Cross-sectional	Moderate	4	No	No	Yes	Yes	Yes	No	Yes	Unclear			
	Qualitative		3	No	No	Yes	Unclear	Yes	No	No	Unclear	Unclear	Yes	

## Appendix 3 Explanation of barriers and facilitators to mental health screening

### Barriers

Figure 3

#### Language and communication

Health care providers expressed various concerns regarding language and communication. Two studies observed that women of refugee backgrounds may use different expressions to describe maternal depressive symptoms, which could impact the accuracy of screening results (Fritz and McGregor 2013; Nithianandan et al. 2016). The availability of translated and validated screening instruments in the mother's native language is important (Skoog et al. 2017). Refugee women prefer to complete the screening in their native language to maintain privacy and avoid the involvement of their husbands or interpreters (Willey et al. 2020a; Willey et al. 2020b). While the translation of screening instruments is deemed important, one study warned of the complexity of translating existing instruments into languages that differ from the dominant (Western) culture (Fritz and McGregor 2013). Several studies identified concerns related to translated versions of specific screening instruments which are delineated under the respective headings.

#### Confidentiality

Both migrant women and health care providers identified concerns about both professional and informal interpreters breaking confidentiality as a potential barrier for women to disclose symptoms of mental health disorders (Nithianandan et al. 2016; Smith 2016; Stapleton et al. 2013; Willey et al. 2020b). In one study migrant women expressed a reduction in their confidentiality concerns when they trusted the interpreter's professionalism (Nithianandan et al. 2016).

#### Stigma

Stigma is a concern for health care providers and migrant women in maternal mental health screening for migrant women. Many studies show that mental health disorders are associated with shame and guilt in migrant women's countries of origin and their communities in the resettling country (Fritz and McGregor 2013; Nithianandan et al. 2016; O’Mahony et al. 2013; Skoog et al. 2019; Skoog et al. 2017; Soldati et al. 2021; Stapleton et al. 2013; Teng et al. 2007; Willey et al. 2020a; Fuggle et al. 2002). This challenges women to speak about their mood with health care providers and might cause them not to give honest answers during mental health screening for the fear of being disregarded by community members or breaking family harmony (O’Mahony et al. 2013; Stapleton et al. 2013; Teng et al. 2007; Willey et al. 2020b). Despite this, in one study women seeking asylum indicated that talking about mental health and seeking professional help was normalized in reception centers, due to the substantial proportion of individuals that face mental health challenges (Soldati et al. 2021).

#### Family support

Studies showed that family can either be a barrier or a facilitator for women to honestly disclose mental health issues (Nithianandan et al. 2016; Soldati et al. 2021; Yu et al. 2019). Women who feared the disapproval of their family or partner were less likely to give honest answers in a mental health screening or accept referrals than women who had their family's support (Nithianandan et al. 2016; Soldati et al. 2021). Yu et al. (2019) found that parents or parent-in-laws had little influence on women's decisions to go for screening because of their lack of knowledge or awareness of PPD (Yu et al. 2019)

#### Practical barriers

Soldati et al. (2021) described practical barriers for women to participate in mental health screening which included proximity to the due date, long waiting times, and difficulties in making an appointment (Soldati et al. 2021).

#### Misconceptions

Two misconceptions women had that prevented them from discussing mental health issues with health care providers were that a referral to psychological care might negatively influence the asylum procedure or that Child Protective Services might take their infant away if a mother revealed that she was mentally unwell (O’Mahony et al. 2013; Skoog et al. 2019; Soldati et al. 2021)

#### Women’s educational attainment and knowledge about mental health

Health care providers commonly believe that migrant women lack an understanding of mental health, which influences the accuracy of screening results (Nithianandan et al. 2016; O’Mahony et al. 2013; Skoog et al. 2017). For example, women might not comprehend the seriousness of mental illness or even understand the concept of PPD as it is not recognized in some cultures (O’Mahony et al. 2013; Skoog et al. 2017). This was confirmed by migrant women in a study where the concept of PPD was unknown to the mothers partially because speaking about mental illness was not common nor accepted in their country of origin (Skoog et al. 2019).

Low educational attainment was recognized as a barrier by health care providers and affected women’s ability to understand and fill out screening questionnaires, particularly the EPDS (Skoog et al. 2017; Smith 2016; Stapleton et al. 2013; Willey et al. 2020b). One study struggled with migrant women’s variable literacy levels and suggested the introduction of audio versions of the screening instrument for these women (Willey et al. 2020a).

#### Health care providers knowledge and cultural competence

While health care providers need skill and knowledge to adequately screen for mental illness during pregnancy, many do not feel well equipped to provide this care to migrant women (Nithianandan et al. 2016; Fuggle et al. 2002; Skoog et al. 2017; Willey et al. 2020b; Fritz and McGregor 2013). Health care providers perceive that they lack knowledge of available screening instruments and mental health disorders. Additionally, health care providers feel like they don't have the required cultural competence to adequately screen for mental health disorders (Nithianandan et al. 2016; Willey et al. 2020b; Skoog et al. 2017).

#### Time constraints

Health care providers identified a lack of time in a busy clinic as a barrier to adequate mental health screening. This includes the lack of time for health care providers to administer screening, but also to address concerns and manage referral in case of a positive screening result (Nithianandan et al. 2016; Kamara 2019; Willey et al. 2020b).

#### Cultural appropriateness of screening instruments

Multiple studies question the cultural appropriateness of screening instruments (Fritz and McGregor 2013; Smith 2016). Most of these instruments were designed in Western cultures and may be inappropriate and ineffective for individuals from cultures with different world views (Fritz and McGregor 2013). For example, the concept of 'self' is not universal, and therefore, women's responses to screening instruments may be influenced by societal expectations in cultures where this concept is absent (Fritz and McGregor 2013). In one study, migrant mothers reported that gender also impacted their ability to express their emotional state, as in some cultures women are not accustomed to receiving this kind of personal attention (Skoog et al. 2019)**.**

### Facilitators

Figure 4

#### Community mental health education

In a study by Nithianandan et al. (2016), health care providers and migrant women recommended community mental health education during pregnancy to increase women’s awareness (Nithianandan et al. 2016).

#### Provider-patiënt relationship

The provider-patient relationship was considered essential for successful maternal mental health screening by migrant women and health care providers (Nithianandan et al. 2016; Skoog et al. 2019; Skoog et al. 2017; Soldati et al. 2021; Willey et al. 2020b). Migrant women expressed that the relationship and quality of contact with their health care providers play a role in their willingness to participate in screening and whether they found it meaningful (Skoog et al. 2019). Trust and continuity of care were considered key in building a strong relationship between patients and their health care providers (Nithianandan et al. 2016; Skoog et al. 2019; Soldati et al. 2021; Willey et al. 2020b). In one study, refugee women expressed that previous negative encounters in care also negatively influenced their willingness to speak about their mood (Skoog et al. 2019).

#### Interpreters

Overall interpreters were considered to facilitate mental health screening especially when screening instruments were not available in women’s native language or in case of low literacy (Skoog et al. 2019; Fuggle et al. 2002). Even if translated instruments were available, interpreters were required to clarify questions and facilitate discussions on mental health between women and health care providers (Fuggle et al. 2002; Willey et al. 2020a; Fritz and McGregor 2013). Engaging interpreters who are appropriately trained and have knowledge about mental health screening is vital to ensure the accuracy of screening results (Willey et al. 2020a; Stapleton et al. 2013). However, in many studies, health care providers described that skills and cultural competence varied between interpreters (Nithianandan et al. 2016; Skoog et al. 2017; Willey et al. 2020a; Fritz and McGregor 2013). To improve consistency, health care providers suggested providing standardized instructions for interpreters regarding EPDS translation, while in another study health care providers kept a list of skilled interpreters they worked with before (Nithianandan et al. 2016; Skoog et al. 2017).

Women were generally content with the quality of interpreters although some considered the interaction with interpreters challenging due to confidentiality concerns and cultural gender roles (Fuggle et al. 2002; Nithianandan et al. 2016; Skoog et al. 2019; Smith 2016; Stapleton et al. 2013; Willey et al. 2020b). To minimize barriers for women, both health care providers and migrant women preferred female on-site interpreters to improve cultural appropriateness (Nithianandan et al. 2016; Skoog et al. 2019).

#### Privacy

Giving women the opportunity to fill in screening instruments in a self-administered form was preferred by migrant women and health care providers in most studies (Willey et al. 2020a; Nithianandan et al. 2016; Fritz and McGregor 2013). In one study, migrant women expressed a preference for health care providers to ask them questions, rather than completing the screening independently. This preference was due to concerns that the written format could be perceived as a test by families experiencing migration issues (Fuggle et al. 2002). Although women preferred to complete the screening in privacy, studies described that in some cases assistance from interpreters or health care providers could be necessary to clarify questions (Yu et al. 2019; Willey et al. 2020a; Fritz and McGregor 2013).

#### Training for health care providers

In four studies health care providers advocated for training staff in mental health and cultural competence to improve screening practices (Kamara 2019; Nithianandan et al. 2016; Skoog et al 2022; Willey et al. 2020b). Two studies evaluated such an educational intervention and reported that all health care providers that participated considered it worth their time and effort (Kamara 2019; Skoog et al 2022)

#### Adequate translation of screening instruments

Adequate translation of screening instruments to ensure cultural sensitivity was considered crucial for the accuracy of screening results (Fritz and McGregor 2013; Nithianandan et al. 2016; Skoog et al. 2017; Yu et al. 2019). Health care providers in a study by Nithianandan et al. (2016) recommended back-translation and gaining community input to achieve cultural equivalence when translating screening instruments (Nithianandan et al. 2016).

## Footnotes

1 For this review, the term migrant will be used as an umbrella term, covering migrants, refugees and asylum seekers, while it is recognized that these groups experience distinct challenges and may have different health needs. For an explanation of the definitions of asylum seeker, refugee, and migrant see box 1 in the methods section. In the results section, we adhered to the terminology used in the included studies as much as possible.
